# Tuberous Sclerosis Associated with Polycystic Kidney Disease: Effects of Rapamycin after Renal Transplantation

**DOI:** 10.1155/2013/397087

**Published:** 2013-01-17

**Authors:** C. Rosado, P. García-Cosmes, P. Fraile, F. Vázquez-Sánchez

**Affiliations:** ^1^Service of Nephrology, University Hospital of Salamanca, Paseo de San Vicente 58-182, 37007 Salamanca, Spain; ^2^Service of Neurology, Public Hospital of Cotentin, Rue Trottebec, 50100 Cherbourg-Octeville, France

## Abstract

Tuberous sclerosis is rarely associated with autosomal dominant polycystic kidney disease in the so-called tuberous sclerosis complex. This association leads to an increased frequency of end-stage renal disease. We present a patient suffering from both syndromes, who received a renal graft and anticalcineurinic drugs as immunosuppressive agents. Progressive titration of the drug was necessary in order to attain the effective doses due to the enzymatic induction caused by concomitant treatment with antiepileptic drugs. These high doses resulted in nephrotoxicity. Immunosuppressor treatment was switched to rapamycin, whereby an improvement in renal function and other signs of tuberous sclerosis and polycystic kidney disease was observed. This case report highlights both the efficacy and safety of rapamycin as an immunosuppressor treatment and its capacity for controlling other symptoms of these genetic-related disorders.

## 1. Introduction

Tuberous sclerosis is a multisystemic genetic disease. Overexpression of mTOR gene is the cause of a wide range of tumours that leads to end-stage renal disease by kidney involvement. Renal polycystosis forms if the patient reaches adulthood due to the development of multiple cyst, angiomyolipomas, and carcinomas in renal parenchyma [1].

Good outcomes under treatment of mTOR inhibitors have been previously reported.

We present a 30-year-old male with end-stage renal disease secondary to tuberous sclerosis and renal polycystosis. After treatment with rapamycin for immunosuppression there was a marked improvement of renal function, tuberous sclerosis manifestations, and renal polycystosis.

## 2. Clinical Case

**Figure 1:**
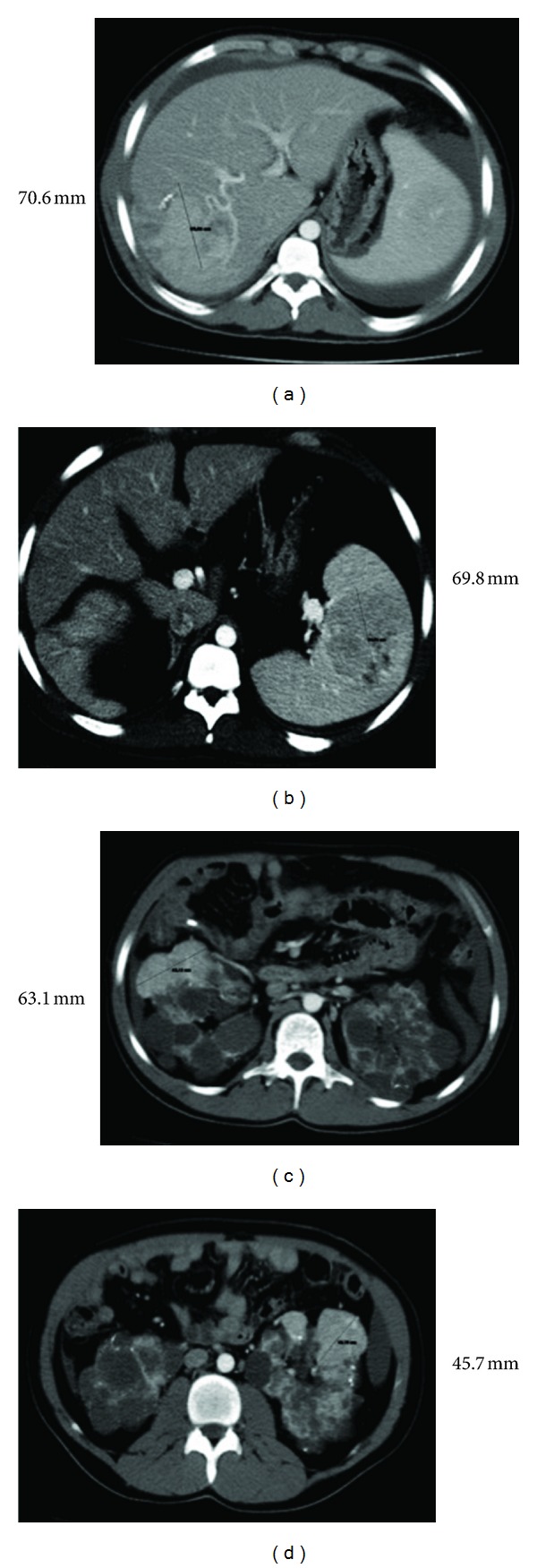
Abdominal CT (prior to renal transplantation) showing different lines of tumours in liver, spleen, and kidneys.

We present a 30-year-old male diagnosed with both tuberous sclerosis (TE) and autosomal dominant polycystic kidney disease. Clinical manifestations began a few months after his birth. He was also suffering from mental retardation, cardiac rhabdomyoma, and various other tumours (angiomyolipomas, hamartoma, cysts, and angiofibroma) including the liver, spleen, and kidneys (Figure 1). He had ungual and cutaneous fibromas and was further afflicted by complex partial seizures controlled with phenobarbital and carbamazepine.

He suffered from end-stage renal disease, and this had been treated with peritoneal dialysis from the age of 24. Two years later he received a renal cadaveric graft from a donor of the same age with two common HLA identities and reached a serum creatinine of 132.6 *μ*mol/L.

Immunosuppression was initiated with baxilisimab, steroids, tacrolimus, and mycophenolate mofetil (this last drug was withdrawn 7 days after treatment began due to persistent diarrhoea). After one month he suffered from an acute graft rejection grade IIa. After being treated with 6-metilprednisolona (6 bolus of 250 mg each), serum creatinine remained around 265.2 *μ*mol/L. Increments of anticalcineurinic drugs, up to 22 mg per day (plasma levels between 6 and 8 ng/mL), were necessary, presumably due to enzymatic induction caused by antiepileptic drugs. Nephrotoxicity, secondary to high doses of anticalcineurinic drugs, was suspected as the cause of renal failure. A switch from anticalcineurinics to rapamycin was made 70 days after the kidney transplantation, starting with 3 mg per day. This was gradually increased and maintained at a level of 6 mg per day (plasma levels 4 to 6 ng/mL). Serum creatinine levels rapidly decreased to the present levels of 114,92 *μ*mol/L. The patient currently remains with a proteinuria of 0.6 g per day and a slight dyslipidaemia.

**Figure 2:**
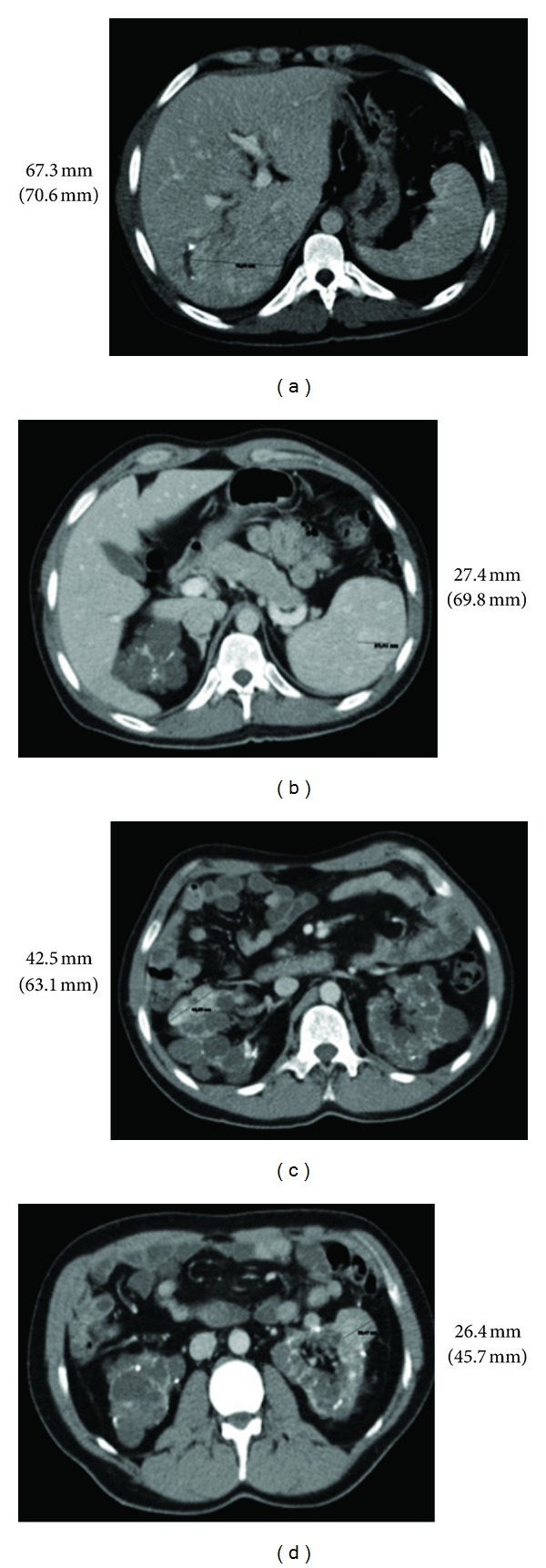
Abdominal CT performed after two years of treatment with rapamycin, showing a significant reduction in the size of tumours.

Since the change to rapamycin, the signs of TE in our patient have diminished remarkably, including a significant reduction in both cutaneous lesions and seizures. This allowed for a gradual reduction of antiepileptic drugs until their complete withdrawal. Furthermore, it also led to the disappearance of cardiac rhabdomyoma and other tumours (Figure 2).

## 3. Discussion

Tuberous sclerosis is a multisystemic autosomal dominant disease that affects 1 : 6000 births. *De novo* mutations make up for about 75% of this total. Its origin lies in mutations of two different tumour suppression genes: TSC1 (9q34), which encode hamartin, and TSC2 (16p13) encoding for tuberin. The tuberin-hamartin protein complex inhibits mTOR (a key protein in the regulation of cell cycle) through the so-called S6 ribosomal kinase plus eIf4EEI- and 4E-binding protein [2] (suppressors of the initiation factor for protein synthesis). Mutations in any of the TSC genes lead to mTOR activation and trigger an uncontrolled proliferation, differentiation, and cellular migration, resulting in different cell line tumours, as well as an increase of the vascular endothelial growth factor (VEFG) contributing to angiogenesis and the growth of tumours.

Typical manifestations of the disease include facial angiofibroma, hypomelanic macules, ungual fibromas, cardiac rhabdomyomas, visceral angiomyolipomas with hepatic predominance, lymphangioleiomyomatosis in women, subependymal astrocytomas, epilepsy, and mental retardation.

Renal angiomyolipomas are present in about 80% of patients, and renal bleeding, arterial hypertension, and chronic kidney disease are the most common complications. Renal substitutive therapy is only required in 1% to 3% of patients due to premature death caused by neurological complications of the disease.

About 2% to 3% of patients carrying a TSC2 mutant gene will also have a grave form of autosomal dominant polycystic kidney disease caused by a deletion in a segment of the pkd1 and TSC2 genes known as contiguous gene syndrome [3].

The hyperactivity of mTOR is a key factor in the pathophysiology of tuberous sclerosis, and its inhibition can be of benefit with regard to tumour development and recurrence [4].

Rapamycin is an immunosuppressor and an antiproliferative agent with the capacity for blocking mTOR linkage to the intracellular receptor FK506-binding protein 12, giving the *phosphorylation *of S6Ks and 4EBP1. This blockage decreases protein synthesis, prohibits cell cycle, and reduces tumoral angiogenesis through the reduction of VEFG levels. Regression of renal angiomyolipomas, astrocytomas, facial fibromas, and lymphangioleiomyomatosis, improvement of epileptic seizures, and the increase of lymphocytic production of cytokines are due to the effect of rapamycin. The effects of rapamycin improve alterations of the immune response inherent to TE [5].

Patients such as the one described previously are good candidates for kidney transplantation. Immunosuppression through mTOR inhibition can improve the prospects due to the regression of tumours and their associated complications [6].

## 4. Conclusions

Our clinical report shows that rapamycin is a good alternative as an immunosuppressant in patients with tuberous sclerosis receiving a renal graft. It demonstrates the advantages with respect to other immunosuppressant drugs, that is, regression of tumoral lesions and improvement of renal cysts. It would also indicate a better pharmacokinetic profile in the concomitant use of inductive drugs frequently used for the treatment of various symptoms of the disease needed in the aforementioned patients. We conclude that rapamycin should be considered as a first-line option in patients with tuberous sclerosis.
